# Machine learning approach to predict 1-year mortality after heart transplantation: a single-centre study

**DOI:** 10.1093/ehjimp/qyaf100

**Published:** 2025-11-07

**Authors:** Batol Allehyani, Maria Teresa Savo, Adel Khwaji, Naji Al Kholaif, Domenico Galzerano, Jehad Al Buraiki, Bandar Alamro, Hani Al Sergani, Giovanni Di Salvo, Dan Alexandru Cozac, Valeria Pergola, Feras Khaliel

**Affiliations:** Heart Centre, King Faisal Specialist Hospital & Research Centre, Makkah Al Mukarramah Br Rd, Al Mathar Ash Shamali, Riyadh 12713, Saudi Arabia; Department of Cardio-Thoraco-Vascular Sciences and Public Health, University of Padua, Padua, Italy; Department of Computer Science, University of Technology, Sydney, Australia; Heart Centre, King Faisal Specialist Hospital & Research Centre, Makkah Al Mukarramah Br Rd, Al Mathar Ash Shamali, Riyadh 12713, Saudi Arabia; College of Medicine, Alfaisal University, Al Takhassousi، Al Zahrawi Street interconnecting Arab Riyadh 11533, Saudi Arabia; Heart Centre, King Faisal Specialist Hospital & Research Centre, Makkah Al Mukarramah Br Rd, Al Mathar Ash Shamali, Riyadh 12713, Saudi Arabia; College of Medicine, Alfaisal University, Al Takhassousi، Al Zahrawi Street interconnecting Arab Riyadh 11533, Saudi Arabia; Heart Centre, King Faisal Specialist Hospital & Research Centre, Makkah Al Mukarramah Br Rd, Al Mathar Ash Shamali, Riyadh 12713, Saudi Arabia; Heart Centre, King Faisal Specialist Hospital & Research Centre, Makkah Al Mukarramah Br Rd, Al Mathar Ash Shamali, Riyadh 12713, Saudi Arabia; College of Medicine, Alfaisal University, Al Takhassousi، Al Zahrawi Street interconnecting Arab Riyadh 11533, Saudi Arabia; Heart Centre, King Faisal Specialist Hospital & Research Centre, Makkah Al Mukarramah Br Rd, Al Mathar Ash Shamali, Riyadh 12713, Saudi Arabia; Division of Pediatric Cardiology, Department for Women’s and Children’s Health, University of Padua, Padua, Italy; Physiology Department, George Emil Palade University of Medicine, Pharmacy, Science and Technology of Targu Mures, Targu Mures 540142, Romania; Heart Centre, King Faisal Specialist Hospital & Research Centre, Makkah Al Mukarramah Br Rd, Al Mathar Ash Shamali, Riyadh 12713, Saudi Arabia; Department of Cardio-Thoraco-Vascular Sciences and Public Health, University of Padua, Padua, Italy; Heart Centre, King Faisal Specialist Hospital & Research Centre, Makkah Al Mukarramah Br Rd, Al Mathar Ash Shamali, Riyadh 12713, Saudi Arabia; College of Medicine, Alfaisal University, Al Takhassousi، Al Zahrawi Street interconnecting Arab Riyadh 11533, Saudi Arabia

**Keywords:** heart transplantation, machine learning, mortality prediction, support vector machine, logistic regression

## Abstract

**Aims:**

Heart transplantation is a critical life-saving procedure for patients with end-stage heart failure. However, predicting postoperative mortality remains challenging. The aim of this study is to examine the effectiveness of machine learning (ML) models for predicting 1-year mortality among heart transplant recipients in Saudi Arabia.

**Methods and results:**

A retrospective observational study was conducted using data from King Faisal Specialist Hospital & Research Centre, a large tertiary hospital in Saudi Arabia, that included all heart transplant cases from January 2007 to December 2022. We evaluate and compare the accuracy of support vector machine (SVM) and logistic regression (LR) models in predicting 1-year mortality. We also identify key predictive variables influencing mortality rates among recipients. SVM and LR models were developed and compared using accuracy, precision, recall, F1 score, and area under the receiver operating characteristic curve as performance metrics. The study analysed data from 419 patients, revealing that ischaemia time, devices like left ventricle assist device, extracorporeal membrane oxygenation, and body mass index (BMI) were significant mortality predictors. The LR model showed a testing accuracy of 96.43%, with weight and BMI having the strongest influence on mortality prediction. The SVM model had a testing accuracy of 95.24%, demonstrating consistent performance across dataset.

**Conclusion:**

The findings indicate that ML models, particularly SVM and LR, are effective in predicting 1-year mortality post-heart transplantation as well as identifying significant predictors of mortality. This research contributes to the global knowledge in heart transplant and highlights the importance of new technologies in tailoring healthcare strategies for the Saudi population.

## Graphical Abstract

**Graphical Abstract:**
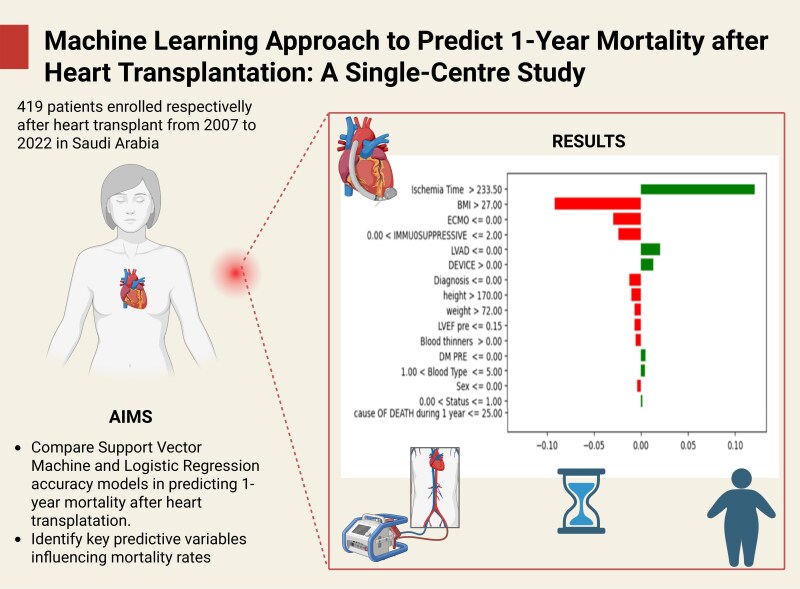

## Introduction

Heart transplantation is a life-saving procedure for patients with end-stage heart failure. However, the success of a heart transplant depends on several factors, including the patient’s overall health, the quality of the donor’s heart, and the patient’s ability to comply with the post-transplant medication regimen. The one-year survival rate after heart transplantation is a commonly used measure of the success of the procedure. Approximately 5000 heart transplants are performed every year and at least 50 000 patients are expected to need heart transplants worldwide every year.^1^ Even though there has been a relatively high success rate in heart transplantation, the limited number of donors constitutes a hindrance that presents a high demand compared with the supply. In other words, there are more potential recipients than donors. Consequently, it is important to consider the risk stratification, which means considering the potential complications of heart transplantation and the complexity of the procedure while scrutinizing the eligibility of patients.^2,3^

Traditionally, prior models have been used to predict risk after heart transplantation. Examples of these types of models are the donor risk index, risk stratification score, independent mortality prediction after cardiac transplantation, and the international heart transplant survival algorithm.^4^ However, their discriminatory capability is modest, which constitutes the main limitation of these models. The development of machine learning (ML) is proving to be a valuable tool for predicting surgical outcomes in contrast to regression-based approaches.^5^ Comparing ML algorithms with logistic regression (LR), ML algorithms have been shown to significantly enhance predictions of outcomes following neurosurgery.^6^ ML techniques have been used in several studies in the past few years to improve heart transplant outcome prediction in adults or combined paediatric and adult populations.^7^

A study by Zhou *et al*.^6^ used several ML algorithms to predict 1-year mortality after heart transplantation. The study compared the performance of several algorithms, including LR, a simple and interpretable algorithm, that is widely used in medical studies. It is based on the logistic function and estimates the probability of the outcome. Additionally, K-nearest neighbours (K-NN), a non-parametric algorithm that can be used for both classification and regression problems was also studied. It found that the random forest algorithm had the best performance for predicting 1-year mortality, with an accuracy of 0.82, followed by decision tree with an accuracy of 0.81, K-NN with an accuracy of 0.78, support vector machine (SVM) with an accuracy of 0.75, and LR with an accuracy of 0.8. The most effective variable for predicting 1-year mortality was the patient’s age at the time of transplantation.

The study also used feature selection to identify the most effective variables for predicting 1-year mortality.

Kampaktsis *et al*.^7^ used a CatBoost algorithm to predict mortality after heart transplantation using a dataset of 1033 patients. The study found that the CatBoost algorithm had an accuracy of 80% in predicting 1-year mortality. They used a total of 49 variables, including demographic variables like age and gender, medical history, pre-transplant laboratory values, donor characteristics, and surgical data. Their findings demonstrate that haemodialysis, after heart transplant, had the strongest relative impact on 1-year mortality of the recipient, followed by the recipient’s estimated glomerular filtration rate, the recipient’s age, and the time since the recipient was ischaemic.

Moreover, Miller *et al*.^5^ used a neural network, classification and regression trees, and random forest algorithms to predict 1-year mortality after heart transplantation using a dataset of 2802 patients. The study found that the random forest is the best fit with an accuracy of 74% in predicting 1-year mortality.

In general, these studies have found that ML algorithms can be used to accurately predict the one-year survival rate of heart transplant patients. The decision tree, random forest, SVM, gradient boosting machine, and deep learning algorithms (DLAs) are effective in predicting the one-year survival rate, with the DLAs achieving the highest accuracy rate.

However, the studies have a few limitations; for example, the sample size is relatively small.

The following tables show the variables that were used in the aforementioned studies when building the ML algorithm. The most important variables in the studies were the recipient’s age and creatinine level, while for the donor was the age and reason for death as shown in *Tables 1* and *2*, respectively.

**Table 1:** Variables used in previous studies for recipients

Total	(15)	(14)	(7)	(6)	(16)	(5)	Variables
5	√	√	√	√		√	Age
1				√			Albumin
1						√	Blood type
3		√	√		√		Body mass index
3		√	√	√			Cardiac surgery history
1		√					Cardiomyopathy
1			√				Citomegalovirus seropositive
5	√	√	√		√	√	Creatinine level
3		√	√			√	Days on waitlist
1	√						Diabetes
2					√	√	Diagnosis category
1			√				Epstein–Barr virus seropositive
2		√				√	ECMO at transplant
1	√						Ejection fraction
1					√		Functional status
4	√	√	√			√	Gender
1			√				Hepatitis B virus (HBsAg+)
1			√				Heart failure aetiology
2			√		√		Height
1				√			Haemoglobin
1	√						High blood pressure
1		√					History of dialysis
1			√				HIV seropositive
1				√			Hypertension
1					√		Ischaemic time
1		√					Karnofsky functional
1			√				Level of education
1						√	LVAD at transplant
1				√			Lymphocyte proportion
1				√			Malignancy history
1		√					Mechanical Circulatory
3		√	√			√	Mechanical ventilation
2					√	√	Medical condition
1	√						Platelets
3		√	√			√	Race/ethnicity
2	√			√			Red blood cell count
1	√						Serum sodium
2		√			√		Serum total bilirubin
2	√			√			Smoking history
1			√				Prostaglandins
2		√				√	Use of inotropic agents
1			√				Ventricular assist device
1						√	Weight

ECMO, extracorporeal membrane oxygenation; HBsAg, hepatitis B surface antigen; HIV, human immunodeficiency virus; LVAD, left ventricle assist device.

**Table 2:** Variables used in previous studies for donors

Total	(15)	(14)	(7)	(6)	(16)	(5)	Variables
4		√		√	√	√	Age
1				√			Alcohol abuse
1						√	Blood type
2		√		√			Body mass index
1				√			Creatinine level
3		√		√	√		Cause of death
2		√		√			Drug abuse
2		√				√	Gender
1				√			Hypertension medical therapy
1		√					Height
1				√			Inotropic support
1				√			PCO2
1						√	Recipient blood match level
1				√			Race/ethnicity
1		√					Trauma cerebrovascular
1						√	Weight

We aimed to evaluate and compare the effectiveness of ML models (SVM and LR) in predicting 1-year mortality rates among heart transplant recipients in Saudi Arabia and identify clinical predictors of this outcome.

## Methods

### Study design, setting, and participant

This retrospective, observational study was designed to evaluate the effectiveness of ML models in predicting 1-year mortality among heart transplant recipients. Given the limited annual frequency of heart transplants, the retrospective approach was deemed most appropriate, allowing for the analysis of a comprehensive dataset over an extended period.^8^ The research was conducted at King Faisal Specialist Hospital & Research Centre and all patients who underwent orthotropic heart transplantation recorded between January 2007 and December 2022 were included. Adhering to ethical guidelines, the study received approval from the institutional review board at King Faisal Specialist Hospital & Research Centre. The study protocol conforms to the ethical guidelines of the 1975 Declaration of Helsinki.

### Model selection

During the initial phase of model selection, we evaluated multiple models, including random forest, gradient boosting, and deep learning models. While these models demonstrated high accuracy during testing, they exhibited overfitting tendencies, as indicated by their near-perfect training accuracies (100% for random forest and 99.1% for gradient boosting). Given the relatively small sample size (*n* = 419), these models posed a risk of reduced generalizability.

To mitigate this risk, we selected LR and SVM, which exhibited high accuracy (96.4% and 95.2%, respectively) while maintaining reasonable training accuracies (96.7% and 96.1%), suggesting better generalizability and stability in a clinical predictive setting.

These models were chosen for their proven capabilities in handling such datasets. The present study compared the effectiveness of these models, focusing on their accuracy and reliability. A feature importance analysis was also conducted to identify the most impactful variables influencing the models’ predictions.

### Variables and data management

The dataset of 419 heart transplant patients includes critical variables affecting post-transplant outcomes. Blood type,^9^ a key factor in donor compatibility, influences immunological responses and survival rates. Sex, body mass index (BMI), and pre-existing conditions like cardiomyopathy or coronary artery disease impact surgical risks and recovery. The use of anticoagulants^10^ and mechanical support [e.g. left ventricle assist device (LVAD) and extracorporeal membrane oxygenation (ECMO)]^11^ are indicators of underlying health issues that may complicate post-transplant survival. Variables like left ventricle ejection fraction (LVEF), ischaemia time, immunosuppressive therapy, and diabetes mellitus further determine patient outcomes. At the same time, transplant date and mortality within the first year are crucial for evaluating success. Understanding these factors is essential for improving care and predicting mortality risks.

## Statistical analysis

Initially, the collected data underwent a comprehensive pre-processing phase. This involved cleaning the data to remove any inconsistencies or missing values, normalizing the data for uniformity, and encoding categorical variables where necessary. The pre-processing step was crucial to prepare the dataset for effective analysis by the ML models.

### Model development

Two ML models SVM and LR were developed. Each model was carefully configured with appropriate parameters to suit the nature of the data. The choice of these models was based on their proven effectiveness in various predictive modelling scenarios, especially in medical data analysis.^12^

### Model training and validation

The models were trained on a portion of the dataset and validated on a separate set. This approach, commonly known as split-sample validation, helps in assessing the models’ performance on unseen data, thereby providing insights into their generalization capabilities.^13^ Additionally, k-fold cross-validation was employed, further ensuring the robustness of the model evaluation process. In k-fold cross-validation, the dataset was divided into ‘k’ subsets, with the model being trained on ‘k-1’ subsets and validated on the remaining subset. This process was repeated ‘k’ times with each subset serving as the validation set once.

### Performance metrics

The models’ performance was evaluated using several metrics, including accuracy, precision, recall, F1 score, and the area under the receiver operating characteristic curve (AUC-ROC). These metrics provided a comprehensive view of the model’s predictive power and reliability. Accuracy measured the proportion of total correct predictions, precision reflected the correctness achieved in the positive class, recall (or sensitivity) indicated the model’s ability to detect positive instances, and the F1 score represented the balance between precision and recall. The AUC-ROC, a plot of the true positive rate against the false positive rate, provided an aggregate measure of performance across all possible classification thresholds.

### Feature importance analysis

To understand the influence of different variables on the models’ predictions, a feature importance analysis was conducted. This analysis identified which variables were most predictive of 1-year mortality, providing valuable insights into the factors impacting patient outcomes post-heart transplantation.^7^

## Results

### Baseline clinical characteristics

In this research, a comprehensive analysis was conducted of the baseline characteristics of heart transplant recipients (*Table 3*), integrating both continuous and categorical variables. This multifaceted approach has allowed for a deeper understanding of the patient demographics and clinical attributes within the study group.

**Table 3:** Baseline clinical characteristics of the heart transplantation population

Parameter	*n* = 419 patients
Demographic characteristics	
Age (years)	33.61 ± 18.94
Male gender (*n*, %)	305 (72.7%)
Weight (kg)	59.2 ± 22.6
Height (cm)	158.5 ± 23.0
Body mass index (kg/m^2^)	22.5 ± 6.4
Types of cardiomyopathies	
Congenital heart disease (*n*, %)	11 (2.8%)
Chemotherapy-induced cardiomyopathy (*n*, %)	3 (0.8)
Dilated cardiomyopathy (*n*, %)	240 (62.9%)
Familial cardiomyopathy (*n*, %)	3 (0.8%)
Hypertrophic cardiomyopathy (*n*, %)	7 (1.8%)
Ischaemic cardiomyopathy (*n*, %)	71 (16.9%)
Kawasaki disease (*n*, %)	2 (0.5%)
Other heart genetic (*n*, %)	8 (2.1%)
Restrictive cardiomyopathy (*n*, %)	17 (4.4%)
Rheumatic heart disease (*n*, %)	14 (3.6%)
Cardiomyopathies not otherwise specified (*n*, %)	3 (0.8%)
Recipients’ blood type	
A (*n*, %)	105 (25.0%)
A^+^ (*n*, %)	27 (6.4%)
AB (*n*, %)	14 (3.3%)
AB^+^ (*n*, %)	6 (1.4%)
AB^−^ (*n*, %)	1 (0.2%)
B (*n*, %)	81 (19.3%)
B^+^ (*n*, %)	17 (4.0%)
B^−^ (*n*, %)	2 (0.5%)
NA (*n*, %)	1 (0.2%)
O (*n*, %)	141 (33.6%)
O^+^ (*n*, %)	24 (5.7%)
Procedure-related implanted device	
Biventricular assist device (*n*, %)	1 (0.2%)
Berlin heart ventricular assist device (*n*, %)	1 (0.2%)
Cardiac resynchronization therapy with defibrillator (*n*, %)	10 (2.4%)
Extracorporeal membrane oxygenation (*n*, %)	8 (1.9%)
Implantable cardiac defibrillator (*n*, %)	48 (11.5%)
Pacemaker (*n*, %)	3 (0.7%)
Unknown type (*n*, %)	2 (0.5%)
Ischaemic time for heart transplantation procedure (minutes)	199.8 ± 74.1
Participant survival status	
Alive (*n*, %)	243 (58.1%)
Dead (*n*, %)	170 (40.7%)
Unknown status (*n*, %)	6 (1.4%)

The average weight of participants was found to be 59.2 kg, spanning a broad range from as low as 1 kg to as high as 130 kg. Height measurements varied significantly as well, averaging 158.5 cm, BMI of the cohort averaged at 22.5, with extremes ranging from 6 to 63. These figures highlight the diverse physical characteristics of the participants.

Ischaemia time, a critical factor in transplantation, showed an average duration of 199.7 min, with a minimum of 67 min and a maximum of 384 min. This variation in ischaemia time underscores the different clinical scenarios encountered during transplantation.

In terms of blood types, ‘O’ blood type emerged as the most common, accounting for a third of the sample, followed by types ‘A’ and ‘B’.

The gender distribution leaned heavily towards male participants, who made up nearly three-quarters of the study group, while female participants represented just over a quarter.

Dilated cardiomyopathy was the most frequently encountered condition, present in nearly two-thirds of the cases. Other significant conditions included ischaemic cardiomyopathy and rheumatic heart disease.

The use of medical devices such as ECMO, implantable cardiac defibrillator, and LVAD was not common, with a significant majority not requiring such devices. ECMO usage was particularly low, indicating its application in more critical cases.

In terms of survival, just over half of the participants were alive at the end of the study period and the causes of death varied, with multi-organ failure, cardiac arrest, and graft failure being among the most common reasons.

### ML model performance

Each model’s effectiveness in predicting 1-year mortality post-heart transplantation was assessed using various statistical measures, providing a clear understanding of their predictive capabilities.

#### Logistic regression

**Figure 1:**
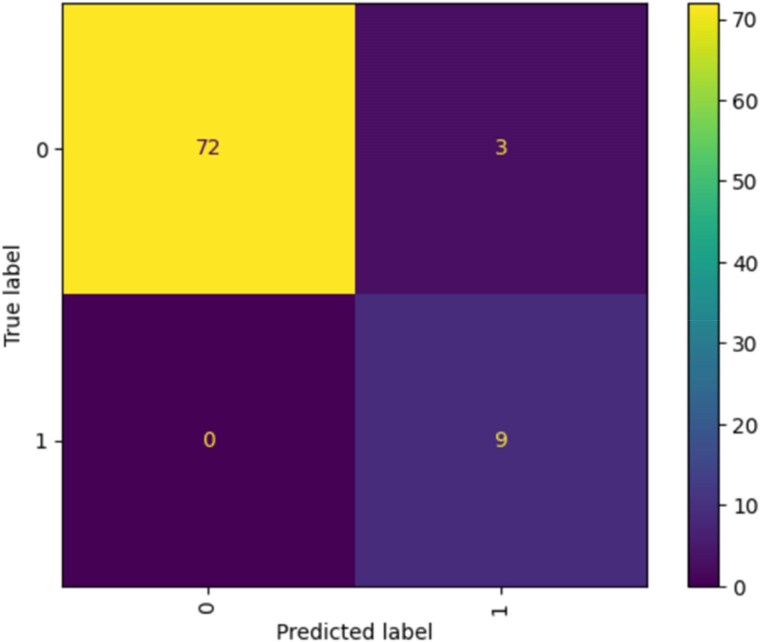
Confusion matrix for logistic regression.

The confusion matrices as illustrated in *Figure 1* provide an integral evaluation of the predictive model’s performance. In analysis, ‘1’ denotes the event of mortality within one-year post-transplantation, whereas ‘0’ signifies survival. The initial confusion matrix revealed that the model accurately predicted survival (true negatives) in 72 instances and correctly identified mortality (true positives) in 9 instances. Conversely, there were three instances where the model failed to predict mortality (false negatives).

**Figure 2:**
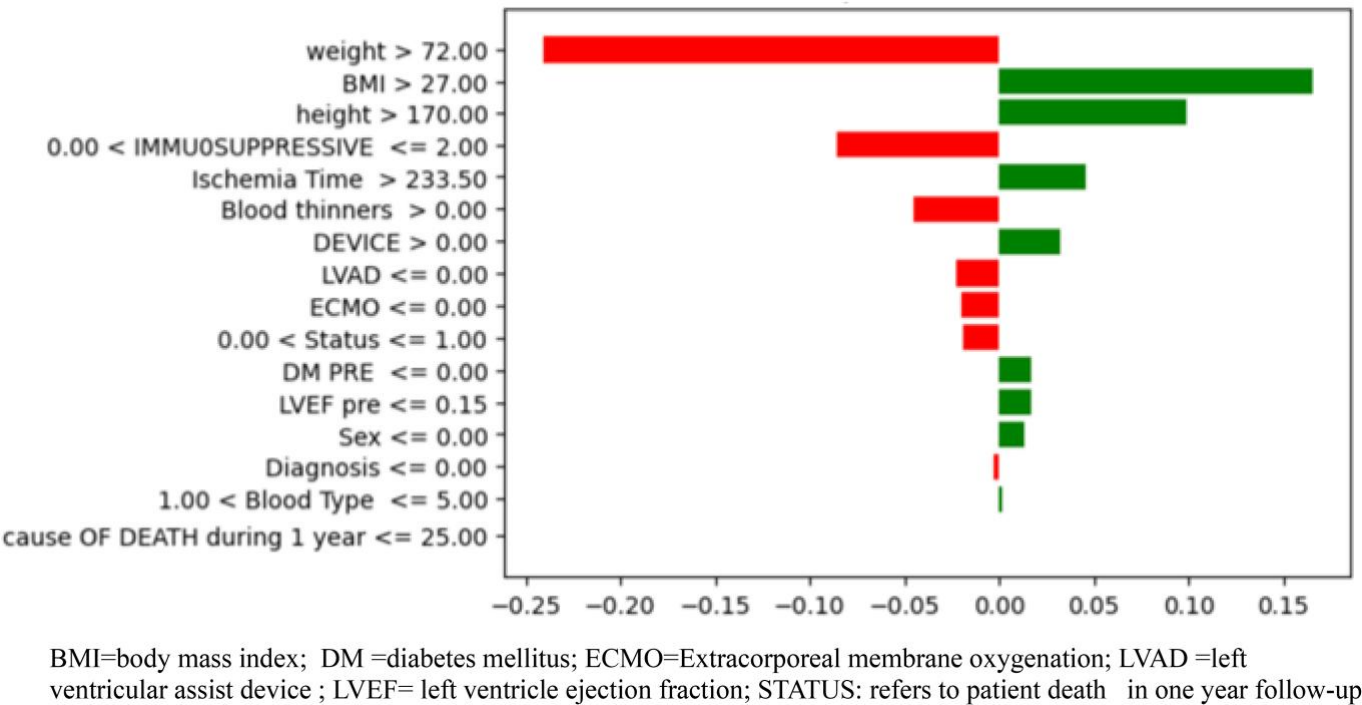
Feature importance plot for predictive model.

The model’s findings as illustrated in *Figure 2* indicate that weight and BMI demonstrate strong statistical associations with mortality outcomes. Specifically, weights above 72 kg and BMI values over 27 kg/m^2^ have been identified as having substantial positive coefficients in the prediction model for one-year mortality. Another notable feature is the ischaemia time, with times over 233.5 min having a moderate positive impact on predicting mortality.

This model showcased excellent performance with a testing accuracy of 96.4% and a perfect precision score of 100%, meaning that it accurately predicted positive instances every time. With a recall of 75.0%, it is robust in classifying positive cases, and an F1 score of 85.7% indicates a strong balance between precision and recall. The model demonstrates good generalizability with an AUC-ROC of 87.5% and a high training accuracy of 96.7%, suggesting that it performs consistently across both training and unseen data.

#### Support vector machine

**Figure 3:**
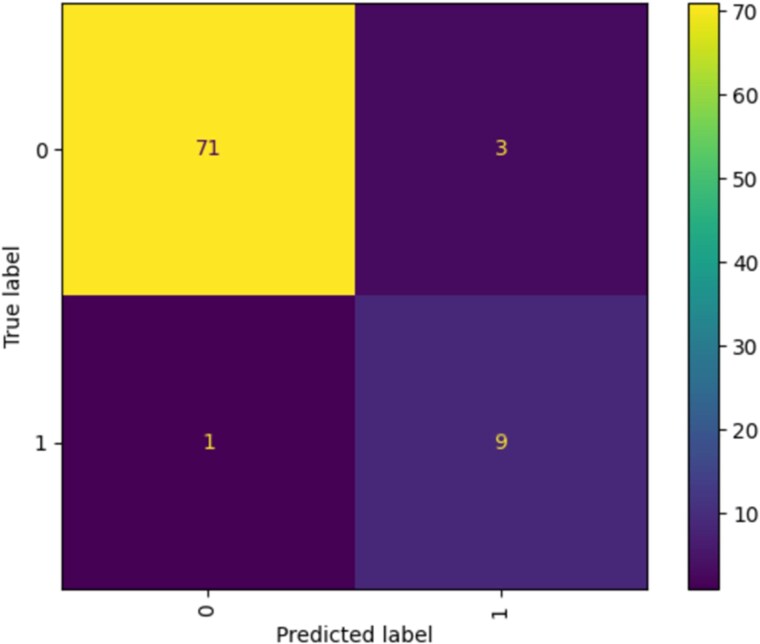
Confusion matrix for support vector machine.

The matrix (*Figure 3*) shows that the model accurately predicted survival in 71 instances (true negative) and correctly identified 9 cases of mortality (true positive). These results indicate that the model is quite adept at identifying patients at high risk of mortality within a year post-transplant, which is critical for early intervention and potentially life-saving measures.

However, the model also predicted survival (false negative) in one case where the patient did not survive. This misclassification highlights an opportunity for further refinement of the model to enhance its predictive accuracy. Additionally, there were three instances where the model incorrectly predicted mortality (false positive).

**Figure 4:**
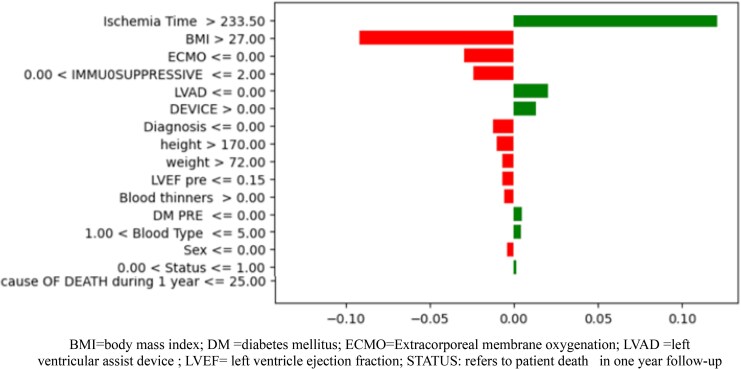
Feature importance in predictive modelling.

The feature importance for the SVM model, as depicted in the local explanation graph (*Figure 4*), highlights the key factors contributing to the prediction of mortality (Class 1) within one year post-heart transplantation.

In this analysis, factors such as BMI above 27 kg/m^2^ and weight above 72 kg are shown to have a strong positive influence on the prediction of mortality, suggesting that higher BMI and weight are prominent indicators associated with the risk of death within the first year following a heart transplant.

Conversely, the model suggests that certain features such as the use of blood thinners and the presence of devices like LVAD or ECMO are negatively correlated with mortality predictions.

Interestingly, the feature of ischaemia time, which is a clinically significant consideration, shows a minimal influence on the model’s predictions for mortality. This could indicate that within the scope of the data and the model, other variables might be capturing the risk associated with prolonged ischaemia time, or that ischaemia time is not as critical a predictor of mortality as other factors in this model.

The model achieved a high testing accuracy of 95.2%, indicating a reliable predictive performance. It demonstrated a high precision of 90.0%, signifying a low false positive rate. With a recall of 75.0% and an F1 score of 81.8%, the model maintains a good balance between sensitivity and precision. The AUC-ROC score of 86.8% and a close training accuracy of 96.1% imply that SVM is effective at distinguishing between classes and is consistent across different dataset.

To ensure a comprehensive evaluation of model performance, we included additional metrics beyond accuracy and precision. These metrics provide a more holistic view of model effectiveness in predicting 1-year mortality after heart transplantation.

**Table:** 

Model	Accuracy	Precision	Recall	F1 score	ROC-AUC	Train accuracy
Logistic regression	0.964286	1.0	0.75	0.857143	0.875	0.967164
Support vector machine	0.952381	0.9	0.75	0.818182	0.868056	0.961194

## Discussion

The main findings of the present study can be summarized as follows: (i) both LR and SVM models identified higher body mass indicators, specifically weights above 72 kg and BMI values over 27 kg/m^2^ as being associated with increased mortality risk within one year following heart transplantation. These findings suggest a significant correlation between elevated body mass and mortality; (ii) the use of blood thinners and the presence of cardiac devices like LVAD or ECMO are associated with a reduced risk of mortality in post-heart transplant patients in the first year of the follow-up; and (iii) notably, despite its clinical importance, the ischaemia time appears to have a minimal impact on the mortality predictions in these models.

This research demonstrates the effective use of LR and SVM to predict one-year mortality after heart transplantation. These findings are in line with existing studies, such as those by Zhou *et al*.^6^ and Kampaktsis *et al*.,^7^ which have explored various ML models. While Zhou *et al*.^6^ utilized algorithms like random forest, the present study adds value by showcasing the effectiveness of LR and SVM, thus broadening the scope of ML applications in heart transplant prognostics.

The high testing accuracies of LR (96.4%) and SVM (95.2%) illustrate the immense predictive power of these models. This echoes the findings of Miller *et al*.,^5^ who highlighted the potential of ML in enhancing the accuracy of medical predictions. The consistency of high performance across different studies and models reaffirms the reliability of ML in clinical settings. The superior performance of the LR model in the study, with an accuracy of 96.4% and a precision of 100%, aligns with broader trends in ML research. Similar studies, such as the one by Zhou *et al*.,^6^ highlight that simpler models like LR can often yield better results in specific scenarios, especially when dealing with datasets that may not be large or complex enough to warrant more sophisticated models. This observation is crucial in the realm of medical predictions, where the balance between model complexity and dataset compatibility plays a significant role in achieving reliable and accurate predictions. The findings of Bhowmik *et al*.^14^ revealed that LR models have outperformed RF and SVM, with the highest ROC-AUC score, emphasizing the effectiveness of simpler models in medical applications. Furthermore, the superiority of simpler models is further supported by research in breast cancer diagnosis , where LR outperformed decision trees and SVMs, offering better interpretability and computational efficiency.^15^ However, a large-scale study using routine clinical data found that more complex algorithms like neural networks outperformed LR in cardiovascular risk prediction, improving accuracy by 3.6% compared with established methods.^16^ The LR model’s ability to efficiently handle the dataset characteristics, while maintaining high accuracy and precision, underscores its suitability for medical predictive tasks, where interpretability and model simplicity are important. Such findings reinforce the idea that in predictive modelling, especially in healthcare, the choice of the model should be tailored to the specificities of the dataset and the clinical context, rather than defaulting to more complex algorithms.

The prominence of BMI and weight as significant predictors in the study models is a recurring theme in heart transplant mortality prediction research. In our study, BMI was used as a proxy for nutritional status, as more detailed nutritional assessments were not available due to the retrospective nature of the dataset. For instance, the studies by Ayers *et al*.^17^ and Özbay Karakuş and Er^18^ have similarly identified physiological parameters as crucial indicators. This study reinforces the importance of these variables, suggesting universal applicability across different populations and healthcare settings. However, more sophisticated nutritional assessment tools may offer enhanced prognostic value in transplantation outcomes. The nutritional risk index, calculated using serum albumin and weight loss, has demonstrated significant prognostic value in surgical and medical patients . Similarly, body composition analysis using techniques such as bioelectrical impedance analysis, dual-energy X-ray absorptiometry, or computed tomography at the L3 vertebral level can provide a detailed assessment of muscle mass, muscle quality, and visceral adiposity.^19^ Emerging research suggests that sarcopenia and myosteatosis may be superior predictors of post-liver transplantation complications compared with BMI alone.^20^ Specifically, reduced skeletal muscle index and increased intramuscular adipose tissue have been associated with prolonged hospital stays, increased infection rates, and higher mortality in various transplant populations.^21,22^.

The inclusion of ischaemia time in the LR model as a predictive variable resonates with findings from Dolatsara *et al*.,^23^ who also emphasized surgical factors. This aspect is crucial, as it highlights the multifaceted nature of heart transplant outcomes, extending beyond patient-specific variables to include procedural elements.

The model’s indication that ischaemia time, specifically times exceeding 233.5 min, has a moderate positive impact on predicting mortality aligns with established medical understanding. This reflects the critical nature of ischaemic time in heart transplantation surgeries. Prolonged ischaemic time is known to increase the risk of complications, which can adversely affect post-transplant outcomes, including mortality. The observation that features like the use of blood thinners and the presence of devices such as LVADs or ECMO are negatively correlated with mortality predictions might be due to these interventions often being associated with more complex, high-risk cases.^24^ These patients might already be at a higher risk, irrespective of the ischaemic time, due to their underlying severe cardiac condition. Thus, while these interventions are necessary and life-saving, they are indicators of a more critical patient profile. However, their use is correlated with a reduction in mortality predictions, suggesting that patients who receive these advanced treatments have a better chance of survival compared with those who do not receive them. While LVAD and ECMO are typically employed in patients with advanced heart failure or severe cardiogenic shock—conditions associated with high baseline mortality—their presence in our dataset is associated with a reduction in predicted mortality. This initially counterintuitive finding likely reflects the life-sustaining role these interventions play in bridging critically ill patients to transplantation. Physiologically, LVADs continuously drain blood from the left ventricle (LV) and deliver it to the aorta, reducing LV size and filling pressures, native stroke volume, and work.^24^ Similarly, ECMO provides temporary cardiopulmonary support, decreasing right ventricle preload, pulmonary blood flow, left ventricular (LV) end-diastolic pressure, and LV end-diastolic volume.^25^ By decreasing central venous pressure, blood flow to organs in the portal circulation improves allowing time for recovery or stabilization before definitive therapy, such as transplantation. By improving haemodynamic stability and end-organ function pre-transplant, these devices may improve perioperative resilience and post-transplant outcomes.

Additionally, the use of such advanced support modalities often occurs in high-volume centres with specialized multidisciplinary teams, which may contribute to better surgical techniques, perioperative care, and post-transplant management—factors known to influence outcomes independently of patient baseline risk. Furthermore, patients selected for LVAD or ECMO support are typically under close surveillance, allowing for optimal timing of transplantation and meticulous management of comorbidities such as renal dysfunction, coagulopathy, and infection.

Finally, this observation may also reflect a form of selection bias: patients who are stabilized successfully on LVAD or ECMO and survive to transplantation may represent a subgroup with favourable response to intensive care, whereas those who do not respond may not survive to transplant and thus are underrepresented in post-transplant outcome data. This correlation suggests that these interventions are effective in managing conditions that would otherwise have a high mortality risk.

The study’s focus on the Saudi Arabian population addresses a critical gap in the literature, which has predominantly concentrated on Western demographics. This regional perspective provides valuable insights into the demographic and health profile peculiarities of the Saudi population, contributing to a more global and inclusive understanding of heart transplant outcomes.

This context is particularly noteworthy due to its unique demographic characteristics, which include prevalent lifestyle diseases and genetic predispositions. By evaluating ML models in this setting, this study offers a model for how regional specificity can be integrated into global medical research.

Demographically, the central region of Saudi Arabia accounts for 76.7% of organ donations, attributed to the presence of a Mobile Action Donor Team.^26^ Saudi Arabia’s cardiac transplant programme reports 1-year survival rates of 85% and 5-year rates of 75%, comparable to international standards.^27^ One high-volume centre in Saudi Arabia demonstrated excellent 30-day mortality and early survival rates, with 87.4% 1-year and 81.5% 3-year survival.^28^ However, challenges persist , including low donor registration rates, lack of public awareness, and high operational costs.^27^ Addressing these issues could potentially improve cardiac transplantation success and reduce cardiovascular disease burden in the region.

A ML algorithm capable of accurately predicting one-year mortality in a specific population, such as heart transplant patients, holds significant clinical implications. Identifying patients at high risk of mortality allows for intensified monitoring, early intervention, and prioritizing care for these populations. Moreover, it could allow a more targeted donor-recipient matching by considering factors beyond traditional criteria. However, a series of challenges should be taken into consideration when using the ML algorithms. A rigorous validation of the algorithm is essential to ensure its accuracy and reliability in different patient populations. Integration of the algorithm into clinical practice requires careful planning to realize its full benefits.

 

#### Strengths and limitations

A relatively large patient sample was enrolled in this study, allowing for a comprehensive assessment of a wide range of risk factors. Employing a ML algorithm to predict mortality in heart transplant patients can identify intricate patterns and relationships among numerous variables that might be overlooked in traditional statistical models. Furthermore, by accurately assessing individual risk, ML can support personalized treatment plans and improve patient outcomes.

Several potential limitations should be acknowledged. A significant limitation of our current analysis is that weight and BMI variables were not stratified by gender, despite well-established physiological differences in body composition and metabolic profiles between male and female patients. We explored both z-score normalization within each sex group and the inclusion of a weight × sex interaction term. However, these adjustments led to substantial declines in model performance. As sex was already included as an independent feature, these methods introduced redundancy that weakened the predictive contribution of weight. The robust predictive performances of our model, as demonstrated in the confusion matrices, provide valuable prognostic information despite this limitation, identifying also opportunities for methodological refinement. This is a single-centre, retrospective study, with a narrow scope of the ML models, suggesting new perspectives and areas for further research. Moreover, given the observational nature of this study, causal relationships cannot be definitively determined.

## Conclusion

The present study highlights the efficacy of LR and SVM models in predicting one-year mortality among heart transplant recipients in Saudi Arabia, with weight, BMI, and ischaemia time identified as key variables. LR showed slightly higher precision, making it potentially more suitable in scenarios demanding high accuracy. The research fills a gap in global heart transplant literature and underscores the importance of regional factors.

## Data Availability

The data underlying this article will be shared on reasonable request to the corresponding author.

## Lead author biography

Senior consultant, adjunct professor of Cardiac Imaging, Department of Cardiac Thoracic Vascular Sciences and Public Health, Padua University, member of the Collegio dei Docenti of the PhD course of Translational Medicine G.B. Morgagni, Padua University.
